# Effects of Zinc Source and Enzyme Addition on the Fecal Microbiota of Dogs

**DOI:** 10.3389/fmicb.2021.688392

**Published:** 2021-10-13

**Authors:** Ana Margarida Pereira, Margarida R. G. Maia, Carlo Pinna, Giacomo Biagi, Elisabete Matos, Marcela A. Segundo, António J. M. Fonseca, Ana R. J. Cabrita

**Affiliations:** ^1^LAQV, REQUIMTE, ICBAS, Instituto de Ciências Biomédicas Abel Salazar, Universidade do Porto, Porto, Portugal; ^2^Dipartimento di Scienze Mediche Veterinarie, Università di Bologna, Ozzano dell’Emilia, Italy; ^3^SORGAL, Sociedade de Óleos e Rações S.A., Aveiro, Portugal; ^4^LAQV, REQUIMTE, Departamento de Ciências Químicas, Faculdade de Farmácia, Universidade do Porto, Porto, Portugal

**Keywords:** dogs, exogenous enzymes, fecal microbiota, pet food, zinc proteinate, zinc sulfate

## Abstract

Supplemental zinc from organic sources has been suggested to be more bioavailable than inorganic ones for dog foods. However, the bioavailability of zinc might be affected by dietary constituents such as phytates. The present study aimed to evaluate the effects of two zinc sources (zinc sulfate and zinc proteinate) and the addition of a multi-enzymatic complex from the solid-state fermentation of *Aspergillus niger* on end-products of fecal fermentation and fecal microbiota of adult Beagles fed a high-phytate diet. The experimental design consisted of three 4 × 4 Latin Squares with a 2 × 2 factorial arrangement of treatments (*n* = 12 Beagles), with four periods and four diets: zinc sulfate without (IZ) or with (IZ +) enzyme addition, and zinc proteinate without (OZ) or with (OZ +) enzyme addition. Enzyme addition significantly affected Faith’s phylogenetic diversity index, whereas zinc source did not affect either beta or alpha diversity measures. Linear discriminant analysis effect size detected nine taxa as markers for organic zinc, 18 for inorganic source, and none for enzyme addition. However, with the use of a negative binomial generalized linear model, further effects were observed. Organic zinc was associated with a significantly higher abundance of Firmicutes and lower Proteobacteria and Bacteroidetes, although at a genus level, the response varied. The DNA abundance of *Clostridium* cluster I, *Clostridium* cluster XIV, *Campylobacter* spp., Ruminococcaceae, *Turicibacter*, and *Blautia* was significantly higher in dogs fed IZ and IZ + diets. Higher abundance of genus *Lactobacillus* was observed in dogs fed enzyme-supplemented diets. End-products of fecal fermentation were not affected by zinc source or enzymes. An increase in some taxa of the phyla Actinobacteria and Firmicutes was observed in feces of dogs fed organic zinc with enzyme addition but not with inorganic zinc. This study fills a gap in knowledge regarding the effect of zinc source and enzyme addition on the fecal microbiota of dogs. An association of zinc bioavailability and bacteria abundance is suggested, but the implications for the host (dog) are not clear. Further studies are required to unveil the effects of the interaction between zinc sources and enzyme addition on the fecal microbial community.

## Introduction

Zinc is the second most abundant transition metal in the body, and it is required for catalytic, structural, and regulatory functions. It is a component of several enzymes being involved in cell replication and differentiation (National Research Council, 2006). The essentiality of zinc for dogs has long been recognized, and dietary levels are recommended for puppies (11 mg per 1,000 kcal of metabolizable energy (ME)) and adult dogs (15 mg per 1,000 kcal of ME for dogs at maintenance, 24 mg per 1,000 kcal of ME for female dogs in late gestation and peak lactation; National Research Council, 2006). The deficiency of zinc in dogs has been associated with skin lesions (Colombini, 1999), behavioral problems (Soltanian et al., 2016), alterations in the immune system, growth, and development, and lesions in the gastrointestinal tract (Vicente et al., 2013).

Zinc absorption takes place in the small intestine, and its homeostasis is regulated by absorption and pancreatic/intestinal excretions (Wang and Zhou, 2010). Regardless of the zinc status of the dog, the absorption of zinc is determined by its solubility in the intestinal compartment and potentially affected by food constituents, such as phytate (a polyanionic molecule with the capacity to chelate positively charged cations such as zinc, inhibiting the absorption of these compounds) and fiber (Walk et al., 2013). Dietary zinc is supplied from raw ingredients and through additives, and in that sense, the source of zinc (organic *vs*. inorganic) is implicated in the success of supplementation. Inorganic sources dissociate easily in the gastric compartment, being more susceptible to interactions than organic sources that usually preserve bonds with organic molecules and might be absorbed as amino acid chelates (Ashmead, 2012). Moreover, organic sources (e.g., zinc amino acid chelates or proteinates) were reported to be more bioavailable for dogs (Lowe et al., 1994; Lowe and Wiseman, 1998; Wedekind and Lowry, 1998) than inorganic sources (e.g., zinc oxide). Despite the active role of the gut microbiome in the health of the host (Guard and Suchodolski, 2016), the effects of zinc sources on the gut microbiome of dogs have not been studied.

The importance of zinc for gut microbiota was already recognized, as chronic zinc deficiency was associated with a decrease in diversity and expansion of species more viable under zinc-limiting conditions, such as Proteobacteria and *Enterococcus* in broilers (Reed et al., 2015). Another study, performed in weaning piglets, showed that dietary supplementation with 562.5 mg/kg of coated zinc oxide prevented intestinal permeability, alterations that usually result in diarrhea, increasing the antioxidant defense mechanism on the mucosa wall (Dong et al., 2019), although the direct effect on microbial communities was not measured.

Generally, zinc modulates the microbiota by promoting the integrity of the intestinal barrier, reducing inflammation (Gordon and Vaishnava, 2019), aiding the regeneration of the epithelium, and controlling the permeation of pathogens (Usama et al., 2018), with the magnitude of the effects being different between zinc sources. Indeed, a study performed in broilers infected with *Clostridium perfringens* reported that organic zinc (zinc proteinate derived from partially hydrolyzed soy protein) enhanced intestinal integrity and partially attenuated the inflammation in the jejunum and cecal tonsil in comparison with zinc sulfate (Bortoluzzi et al., 2019). In lactating dairy cows, supplementation with organic zinc (glycinate) was more efficient than inorganic zinc in decreasing the relative abundance of fecal *Treponema* spp. (Faulkner et al., 2017), a rumen bacteria associated with bovine digital dermatitis (Zinicola et al., 2015). This highlights the role of zinc in the regulation of the permeability and expansion of pathogens that use the gut as a reservoir.

Moreover, zinc is vital for bacterial physiology, being present in around 6% of their proteome (Capdevila et al., 2016). It is not known if bacterial uptake of zinc is different for free zinc and zinc chelates. However, host defense mechanisms against bacterial infection include zinc sequestration and chelation to withhold it from invading bacteria (Hood and Skaar, 2012).

Due to the effects of dietary antagonists (e.g., fiber and phytate), the addition of exogenous enzymes (containing residual activity of carbohydrases, phytase, and protease) has been suggested as a strategy to increase zinc bioavailability in diets for monogastric animals (Perić and Spring, 2013). Moreover, the addition of exogenous enzymes can also affect the gut microbiota, as shown by Diógenes et al. (2018), who reported differences in community richness and diversity in fish fed diets supplemented with commercial exogenous complexes—a natural solid-state fermentation complex of *Aspergillus niger* with residual enzyme activity or an enzyme complex of highly purified non-starch polysaccharidases.

Recently, our group evaluated the effects of supplementing organic (proteinate) or inorganic (sulfate) zinc sources (75 mg of supplemental zinc) with or without the addition of a commercial multi-enzymatic complex from the solid-state fermentation of *A. niger* to high-phytate-content diets on the zinc status of dogs (Pereira et al., 2020). Higher bioavailability of phosphorus and a potential effect on immune function associated with zinc proteinate were observed, whereas the addition of exogenous enzymes did not affect zinc availability or digestibility of both nutrients and energy (Pereira et al., 2020). The effects of these zinc sources and enzyme addition on gut fermentation profile and microbiota are reported in the current study. For that, end-fermentation products, including volatile fatty acids (VFA), lactate, and ammonia-N, were determined in fecal samples of dogs fed the experimental diets; microbial DNA was extracted, allowing the characterization of the bacterial community profile and the quantification of selected bacterial groups.

## Materials and Methods

The trial was approved by the Local Animal Ethics Committee of Abel Salazar Biomedical Sciences Institute, University of Porto, and licensed by the Portuguese Directorate-General of Food and Veterinary Medicine (permit No. 206/2017). All the procedures were performed by trained personnel (FELASA category C).

### Animals and Diets

The study was conducted according to three independent 4 × 4 Latin Squares with a 2 × 2 factorial arrangement of treatments, with four diets, four consecutive periods (each lasting 5 weeks), and 12 one-year-old Beagles (six males and six females; 11 ± 1.1 kg of body weight (BW); see Supplementary Figure 1), totaling 48 fecal samples analyzed. This design was selected to increase the number of replicas of each treatment with the minimum number of animals, according to the 3R’s policy. The length of each period was defined based on evidence that dogs’ fecal microbiome responds to dietary transition within 4 weeks (Allaway et al., 2020). The four experimental diets (Table 1) differed on the source of supplemental zinc (zinc sulfate for diets IZ and IZ +; and zinc proteinate for diets OZ and OZ +). Also, a commercial multi-enzymatic complex from the solid-state fermentation product of *A. niger* (including phytase, protease, xylanase, β-glucanase, cellulase, amylase, and pectinase; Synergen^®^, Alltech, Nicholasville, KY, United States) was added to diets IZ + and OZ +, and not to IZ and OZ. Supplemental zinc sources provided ≈75 mg/kg of zinc.

**TABLE 1:** Ingredient list (g/kg, wet weight) and chemical composition (units/kg dry matter (DM)) of the experimental diets supplemented with inorganic (IZ and IZ +) and organic (OZ and OZ +) zinc sources without (IZ and OZ) and with (IZ + and OZ +) the addition of a multi-enzymatic complex from the solid-state fermentation of *Aspergillus niger*.

Ingredient				
Wheat bran	188			
Wheat	154			
Poultry by-product meal	117			
Maize	98			
Soybean protein concentrate	94			
Mammal fat	56			
Broken rice	47			
Pea starch	47			
Hydrolyzed salmon protein	47			
Palatability enhancer	38			
NuPro^®^ yeast	28			
Alfalfa protein concentrate	23			
Sugar beet pulp	23			
Premix^1^	14			
*Ascophyllum nodosum*	9.4			
Mono-ammonium phosphate	7.5			
Milled salt	5.0			
Preservative	3.8			
Sodium hexametaphosphate	0.3			
Zinc sulfate mono hydrate (mg/kg)^2^	206			
Zinc proteinate (Bioplex^®^, Alltech; g/kg)^3^	5			
Solid-state fermentation product of *A. niger* (mg/kg)^4^	200			

**Chemical composition**	**IZ**	**IZ +**	**OZ**	**OZ +**

Dry matter, g	922	930	910	911
Ash, g	71.9	69.6	70.9	75.3
Crude protein, g	274	280	288	291
Ether extract, g	120	118	115	111
Neutral detergent fiber, g	191	212	217	207
Starch, g	310	310	287	284
Phytic acid, g	10.6	9.70	11.0	9.70
Zn, mg	159	139	140	150

*^1^Premix per kg of diet: vitamin A, 14,950 UI; vitamin D3, 1,560 UI; vitamin E, 98.0 mg; thiamine, 2 mg; riboflavin, 4 mg; niacin, 30 μg; cobalamin, 30 μg; vitamin B6, 3 mg; folic acid, 495 μg; biotin, 150 μg; vitamin K, 2 mg; pantothenic acid, 20 mg; CuSO_4_ ⋅ 5H_2_O, 8 mg; ıKI, 2 mg; MnSO_4_ ⋅ H_2_O, 5 mg; Na_2_SeO_3_, 100 μg.*

*^2^Only in diets IZ and IZ +.*

*^3^Only in diets OZ and OZ^+^.*

*^4^Only in diets IZ + and OZ +.*

Dogs were individually fed their daily ration in two meals (9:00 a.m. and 5:00 p.m.), which was calculated to meet dogs’ ME requirements (National Research Council, 2006) adjusted to their body condition score (BCS; 9-point scale; Laflamme, 1997).

### Sample Collection

Fresh feces were collected within 1 h of defecation on the last day of each experimental period. Samples were weighed, pooled, and split to be frozen at −80°C for fecal microbiota analysis and −20°C for the remaining analyses.

### pH, Ammonia-N, Lactate, and Volatile Fatty Acid Analysis

Feces were thawed and diluted to 1:10 in water for pH measurement using a potentiometer (pH and Ion-Meter GLP 22, Crison, Barcelona, Spain). Ammonia-N was determined in 1 g of feces diluted in 200 ml of ultrapure water (18.2 MΩ cm; Arium^®^, Sartorius, Göttingen, Germany) and subjected to a gas-diffusion microextraction with *o*-phthalaldehyde labeling for fluorometric determination in a microplate reader (Synergy HT, BioTek Instruments, Bad Friedrichshall, Germany), according to Valente et al. (2017).

For the determination of lactate, 1 g of feces was diluted into 10 ml of ultrapure water and homogenized by vortexing and ultrasound (5 min)-assisted mixing. Samples were then centrifuged for 15 min at 2,415 × *g* and 4°C and filtered using a 0.45-μm pore size polyethersulfone syringe filter (VWR International, Alfragide, Portugal). The supernatant was recovered and assayed using a commercial kit (D-/L-lactic acid, NZYTech, Lisbon, Portugal) adapted to a microplate format. The UV detection was performed using the above-mentioned microplate reader. Lactic acid is presented as the sum of D- and L-lactic acids.

For VFA analysis, 1 g of feces was diluted in 10 ml of 25% ortho-phosphoric acid solution with internal standard (4 mM of 3-methyl valerate, Sigma Aldrich, St. Louis, MO, United States); the mixture vortexed and centrifuged for 60 min at 5,251 × *g*, at 4°C. The supernatant was filtered using a 0.45-μm pore size polyethersulfone syringe filter (VWR International) and analyzed by gas chromatography using a Shimadzu GC-2010 Plus (Shimadzu Corporation, Kyoto, Japan) equipped with a capillary column (HP-FFAP, 30m × 0.25 mm × 0.25 μm; Agilent Technologies, Santa Clara, CA, United States) and a flame ionization detector. Individual VFA was identified by comparison of retention times with a commercial standard and quantified with the internal standard method as described by Maia et al. (2016).

### Microbiota Analysis

#### Sequencing of 16S rRNA Genes

Bacterial genomic DNA was extracted from 200 mg of frozen fecal samples using a stool DNA isolation kit (Norgen Biotek Corp., Thorold, ON, Canada) following the procedure recommended by the manufacturer, including a bead-beating step. Isolated DNA purity and concentration were assessed with a spectrophotometer (DS-11, DeNovix^®^, Wilmington, DE, United States). Total genomic DNA was diluted to 10 ng/μl and sent to StarSEQ (Mainz, Germany), which performed the sequencing of the hypervariable V3–V4 regions of the 16S rRNA encoding gene by a dual-index strategy based on the protocol of Caporaso et al. (2012) and Apprill et al. (2015), with minor modifications.

The sequencing used the F341/R806 primer set (Takahashi et al., 2014) and AccuStart^TM^ II PCR ToughMix^®^ (Quantabio, Beverly, MA, United States) in the reaction. The amplification was performed in a Thermocycler T-Professional (Biometra, Göttingen, Germany) for a 33-cycle PCR amplification step according to Bolyen et al. (2019), with modifications to optimize the overall performance. Amplicons were checked for quality with QIAxcel^®^ capillary electrophoresis (Qiagen, Hilden, Germany), normalized, and pooled for quantification. Over 15% of the PhiX control library was spiked into the amplicon pool to improve the unbalanced and biased base composition. The sequencing primers for forward sense strand (5′-GGCTGACTGACT-3′) and reverse sense strand (5′-CCAATTACCATA-3′) were added to MiSeq Reagent Kit V3 (Illumina, San Diego, CA, United States) as well as a positive control (ZymoBIOMICS^TM^ Microbial Community DNA Standard, Zymo Research Corp., Irvine, CA, United States). The 2 × 300 bp paired-end sequencing was run on a MiSeq platform (Illumina). The sequences from the MiSeq Illumina were analyzed using the QIIME 2 version 2018.6 (Bolyen et al., 2019). Raw reads were de-multiplexed and quality checked by FastQC (Andrews, 2010). Paired-end reads were joined by the tool PEAR. Low-quality reads were removed. Reads were corrected, chimeras were removed, and amplicon sequence variants (ASVs) were obtained by the deblur workflow (Amir et al., 2017). Then, a multiple sequence alignment (Katoh et al., 2002) and a phylogenetic tree were generated (Price et al., 2009). Alpha diversity rarefaction curves were generated for each category [zinc source (Zinc), enzyme addition (Enzyme), and interaction between zinc source and enzyme addition (Zinc × Enzyme)] and each sample. Taxonomy was assigned to ASVs using a naive Bayes approach of the scikit learn Python library (Bokulich et al., 2018) and the SILVA database version 123 (Quast et al., 2013). Interactive stacked bar charts of the taxonomic abundances of each category and each sample were generated. Alpha and beta diversity metrics were calculated after normalization by rarefaction (at the lowest sample size). Alpha diversity metrics were calculated using Shannon’s diversity index and Faith’s phylogenetic diversity (Faith’s PD) to assess the community’s richness and Pielou’s evenness to assess the community’s evenness. Beta diversity metrics calculated were weighted and unweighted UniFrac distances to assess community dissimilarity. The principal coordinates analysis (PCoA) was used to plot the distance matrixes.

#### Quantitative PCR

To further confirm abundances obtained with 16S rRNA gene sequencing, qPCR assays were performed in selected bacterial groups: *Campylobacter* spp., *Blautia* spp., *Turicibacter* spp., Ruminococcaceae, *Campylobacter jejuni*, *Campylobacter lari*, *Escherichia coli*, *Clostridium* cluster I, and *Clostridium* cluster XIV. Detailed information on the primers used is presented in Table 2. Bacterial DNA extracted from each fecal sample was pooled together, diluted at 50 ng/μl and used to test the appropriate annealing temperature for each primer. For this purpose, a gradient qPCR was performed with the following condition: the reaction contain a total volume of 15 μl, of which 1.5 μl of pooled DNA (50 ng/μl), 7.5 μl of 2× SensiFAST No-ROX PCR MasterMix (Bioline GmbH, Luckenwalde, Germany), 4.8 μl of nuclease-free water, and 0.6 μl of each 10 pmol of primers. Amplification and detection were carried out in a CFX96 Touch thermal cycler (Bio-Rad, Hercules, CA, United States) after an initial denaturation of 2 min at 95°C, followed by 40 cycles of 95°C for 5 s, gradient (58°C–66°C) for 10 s, and 72°C for 12 s. PCR products from the primer test were then purified using a commercial kit (QIAquick PCR Purification Kit, Qiagen, Hilden, Germany) following the manufacturer’s instruction. PCR products were quantified with a DS-11 spectrophotometer (DeNovix^®^, Wilmington, DE, United States). The copy numbers of each amplicon were calculated using the formula (DNA concentration (μg/μl) × 6.0233 × 10^23^ copies/mol)/(DNA size (bp) × 660 × 10^6^) (Jin et al., 2018). PCR products were finally diluted 1:1,000 or 1:100 (v/v), according to the number of copies in the original solution, in order to reach a concentration of 10^9^ or 10^8^ copies. A 10-fold dilution series was done for each standard, reaching a final concentration of 10^2^ or 10^1^ copies, depending on the bacterial target. Each dilution step was performed by diluting 5 μl of the stock solution in 45 μl of nuclease-free water (1:10 v/v). Amplification and quantification were carried out in a CFX96 Touch thermal cycler (Bio-Rad, Hercules, CA, United States) after an initial denaturation of 2 min at 95°C followed by 40 cycles of 95°C for 5 s, primer annealing (59°C–64°C) for 10 s, and 72°C for 12 s. Amplification was run in a total volume of 15 μl, 1.5 μl of DNA template of each sample (50 ng/μl), 7.5 μl of 2× SensiFAST No-ROX PCR MasterMix (Bioline GmbH, Luckenwalde, Germany), 4.8 μl of nuclease-free water, and 0.6 μl of each 10 pmol of primers. All samples were determined in duplicate and standards in triplicate. A no-template control (DNase-free water) was run for each primer assay. Cycle threshold values were plotted against standard curves for the quantification of the target bacterial DNA. The copy numbers for microbial populations were calculated with the formula above reported (Jin et al., 2018). Results were reported as log_10_ copies/g feces.

**TABLE 2:** Primers used in the qPCR assay.

Target (amplicon size)	Primer	Sequence (5′–3′)	Annealing temperature (°C)	References
*Campylobacter* spp. (108 bp)	R-campF2	CAC GTG CTA CAA TGG CAT AT	62	Lund et al., 2004
	R-campR2	GGC TTC ATG CTC TCG AGT T		
*Blautia* spp. (250 bp)	BlautiaF	TCTGATGTGAAAGGCTGGGGCTTA	59	Suchodolski et al., 2012
	BlautiaR	GGCTTAGCCACCCGACACCTA		
*Turicibacter* spp. (140 bp)	TuriciF	CAGACGGGGACAACGATTGGA	62	Suchodolski et al., 2012
	TuriciR	TACGCATCGTCGCCTTGGTA		
*Clostridium* cluster I (231 bp)	CloI-F	TACCHRAGGAGGAAGCCAC	62	Song et al., 2004
	CloI-R	GTTCTTCCTAATCTCTACGCAT		
*Clostridium* cluster XIV (157 bp)	CloXIV-F	GAWGAAGTATYTCGGTATGT	59	Song et al., 2004
	CloXIV-R	CTACGCWCCCTTTACAC		
*Escherichia coli* (340 bp)	Coli_F	GTTAATACCTTTGCTCATTGA	59	Malinen et al., 2003
	Coli_R	ACCAGGGTATCTAATCCTGTT		
Ruminococcaceae (258 bp)	Rumi_F	ACTGAGAGGTTGAACGGCCA	64	Garcia-Mazcorro et al., 2012
	Rumi_R	CCTTTACACCCAGTAAWTCCGGA		
*Campylobacter jejuni* (124 bp)	CjejF	TGCACCAGTGACTATGAATAACGA	62	He et al., 2010
	CjejR	TCCAAAATCCTCACTTGCCATT		
*Campylobacter lari* (86 bp)	ClariF	TTAGATTGTTGTGAAATAGGCGAGTT	59	He et al., 2010
	ClariR	TGAGCTGATTTGCCTATAAATTCG		

### Statistical Analysis

QIIME 2 workflow was used to select ASVs, sequence alignment, inferring phylogenetic trees, and phylogenetic and taxon-based analyses of alpha and beta diversity within and between samples as described by Caporaso et al. (2010). Given the non-parametric nature of microbiota data, indices of alpha diversity data were analyzed using the Kruskal–Wallis test. Differences in beta diversity were analyzed using the non-parametric permutational multivariate analysis of variance (PERMANOVA) and the test for homogeneity of multivariate dispersions (PERMDISP), both with 999 permutations. *p*-Values were corrected using the Benjamini–Hochberg false discovery rate (FDR) method.

Per-sample normalization of the sum of the values to 1M was performed, and the effects of Zinc, Enzyme, and Zinc × Enzyme on the relative abundance of taxa were tested using a non-parametric factorial Kruskal–Wallis sum-rank test, followed by linear discriminant analysis (LDA) effect size (LEfSe; Segata et al., 2011).

Fermentation parameters data (fecal pH, ammonia-N, lactate, and VFA) and DNA concentration of selected bacterial groups were subjected to a least-squares ANOVA for three 4 × 4 Latin Squares with a 2 × 2 factorial arrangement of treatments using the generalized linear model (GLM) procedure of SAS (SAS^®^ University Edition 2019, Cary, NC, United States). The model included the fixed effects of the square, dog within the square, period, zinc source (Zinc), enzyme addition (Enzyme), the interaction between zinc source and enzyme addition (Zinc × Enzyme), and the residual error. Counts of taxa present in at least 50% of the samples and with a relative abundance >0.01% were analyzed using a negative binomial GLM (NBGLM; Proc Glimmix of SAS^®^) including the fixed effects previously mentioned. The statistical level of significance was set for *p* < 0.05. Tukey’s test was applied for *post hoc* analysis.

## Results

### QIIME Analysis of Sequencing Data

The total number of sequences obtained after filtering for quality, trimming length, and assigning taxonomy was 4,578,668 from 48 samples with an average of 95,398 ± 31,303 reads per sample (range 25,675–152,153). A total of 2,516 ASVs were identified and 212 assigned to the genus level. After rarefaction and normalizing to the sample with the lowest number of sequences (3,296), 161,504 sequences were retained (24.7%). Rarefaction curves were adequate for the analysis, as they all tended to a plateau (Supplementary Figure 2). The number of observed species per sample ranged from 62 to 212.

**FIGURE 1:**
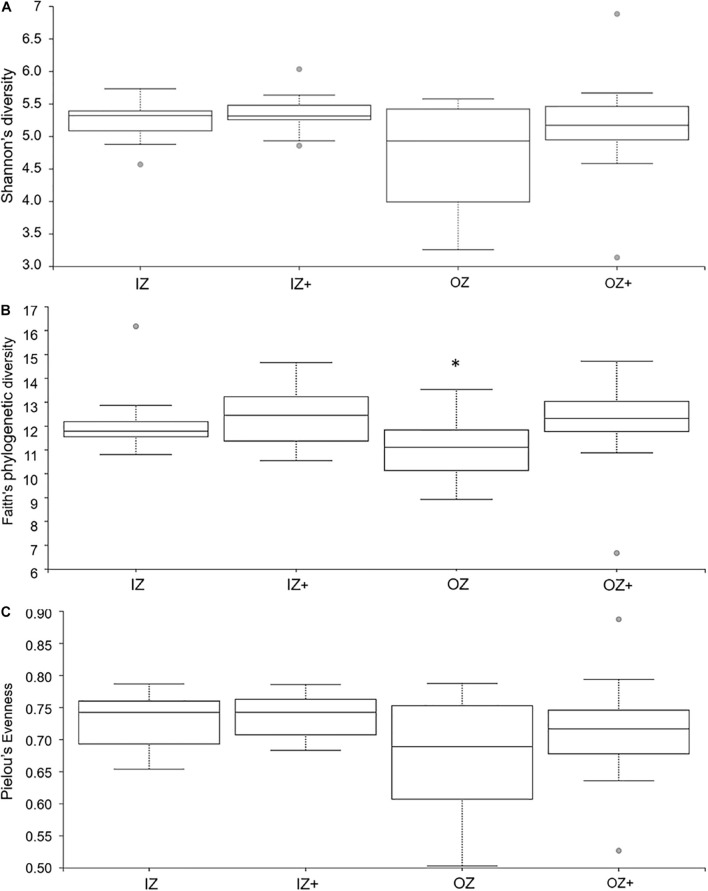
Boxplot of alpha diversity indices calculated for fecal bacteria associated with diets supplemented with inorganic (IZ and IZ +) and organic (OZ and OZ +) Zn sources without (IZ and OZ) and with (IZ + and OZ +) the addition of a multi-enzymatic complex from the solid-state fermentation of *Aspergillus niger.* Boxes represent the interquartile range between the first and third quartiles, and the horizontal line inside the box defines the median. Whiskers represent the lowest and highest values, not considering outliers, represented by the dots. **(A)** Shannon’s diversity, **(B)** Faith’s phylogenetic diversity, and **(C)** Pielou’s evenness. *Statistically significant (*p* < 0.05).

Means of ASVs were 100,317 ± 23,863, 97,961 ± 28,401, 78,753 ± 28,894, and 103,404 ± 40,870 for diet IZ, IZ +, OZ, and OZ +, respectively. Alpha metrics for Zinc × Enzyme are presented in Figure 1. Shannon’s diversity index was not affected by Enzyme (*H* = 0.86, *p* = 0.353), Zinc (*H* = 2.20, *p* = 0.138), and Zinc × Enzyme (*H* = 3.10, *p* = 0.377). Faith’s PD was affected by Enzyme (*H* = 4.00, *p* = 0.045), but not Zinc (*H* = 0.71, *p* = 0.398) and Zinc × Enzyme (*H* = 5.95, *p* = 0.114). The community evenness (Pielou’s evenness) was unaffected by Zinc (*H* = 2.32, *p* = 0.127), Enzyme (*H* = 0.49, *p* = 0.483), and Zinc × Enzyme (*H* = 2.92, *p* = 0.403). Pairwise comparisons of alpha metrics for Zinc × Enzyme are presented as Supplementary Material (Supplementary Table 1); no differences were observed (*q*-value > 0.05).

### Principal Coordinates Analysis

**FIGURE 2:**
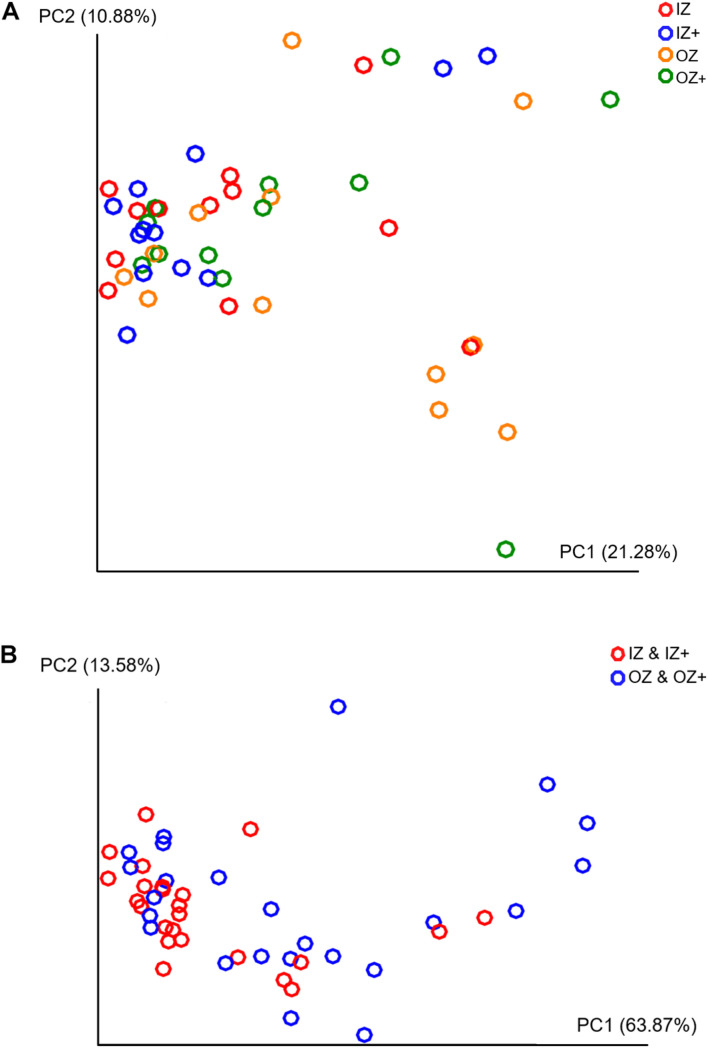
Beta diversity metrics. Principal coordinates analysis (PCoA) of unweighted UniFrac distances of fecal bacteria associated with diets supplemented with inorganic (IZ and IZ +) and organic (OZ and OZ +) Zn sources without (IZ and OZ) and with (IZ + and OZ +) the addition of a multi-enzymatic complex from the solid-state fermentation of *Aspergillus niger* **(A)** and weighted UniFrac distances of fecal bacteria associated with diets supplemented with inorganic and organic Zn **(B)**.

Unweighted and weighted UniFrac distance analyses were performed and projected onto two-dimensional plots using PCoA (Figure 2). Unweighted PCoA showed that samples were more clustered on the principal coordinate (PC) 2 and PC 3, except those from the OZ treatment (Figure 2A), and PERMANOVA on unweighted UniFrac distances showed a significant effect from Zinc (pseudo-*F* = 1.750, *p* = 0.035) and no effects from Enzyme (pseudo-*F* = 0.861, *p* = 0.602) and Zinc × Enzyme (pseudo-*F* = 1.160, *p* = 0.188, Supplementary Table 2). PERMDISP analysis was significant for Zinc (pseudo-*F* = 4.540, *p* = 0.047) and not significant for Enzyme (pseudo-*F* = 0.686, *p* = 0.179) and Zinc × Enzyme (pseudo-*F* = 1.302, *p* = 0.282, Supplementary Table 3).

Similarly, PERMANOVA on weighted UniFrac distance showed no effects from Enzyme (pseudo-*F* = 0.652, *p* = 0.535) and Zinc × Enzyme (pseudo-*F* = 1.840, *p* = 0.108) and a significant effect of Zinc (pseudo-*F* = 4.540, *p* = 0.019, Supplementary Table 4). PERMDISP test was not significant for Zinc (pseudo-*F* = 6.25, *p* = 0.028) and Enzyme (pseudo-*F* = 1.18, *p* = 0.194) but was significant for Zinc × Enzyme (pseudo-*F* = 3.437, *p* = 0.029, Supplementary Table 5). In line with this, Figure 2B exhibits a higher dispersion of samples from dogs fed diets OZ and OZ + along PC1 than of those fed IZ and IZ + diets.

### Fecal Microbiota Profiling

**FIGURE 3:**
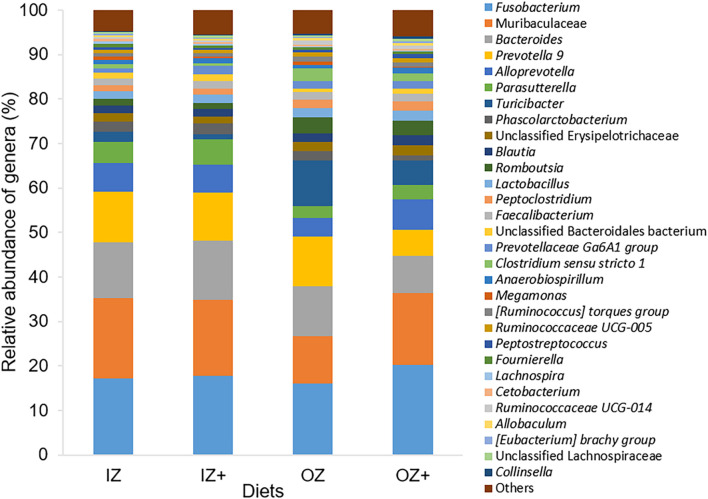
Relative abundance (%) of bacterial genera in fecal samples of dogs fed diets supplemented with inorganic (IZ and IZ +) and organic (OZ and OZ +) Zn sources without (IZ and OZ) and with (IZ + and OZ +) the addition of a multi-enzymatic complex from the solid-state fermentation of *Aspergillus niger*. Genera with relative abundance <0.5% were pooled and named “Others.”

After normalization of sequences into relative abundances, 11 phyla, 15 classes, 34 orders, 62 families, and 212 genera were identified. From those, five phyla, eight classes, nine orders, and 13 families presented relative abundances > 1% and 62 genera > 0.01% (Figure 3). Regardless of the zinc source and enzyme addition, the phyla Bacteroidetes, Firmicutes, and Fusobacteria, the classes Bacteroidia, Fusobacteriia, and Clostridia, and orders Bacteroidales, Fusobacteriales, and Clostridiales were the three most abundant in each taxon. At the family level, Muribaculaceae, Prevotellaceae, Fusobacteriaceae, and Bacteroidaceae were the four most abundant families, irrespective of zinc source and enzyme addition. At the genus level, *Fusobacterium* was the most abundant genus, for all experimental diets. Additionally, *Turicibacter*, [*Ruminococcus*] torques group, *Faecalibacterium*, Ruminococcaceae UCG-005, and *Collinsella* were present in all 48 fecal samples, whereas the remaining genera were not detected in at least one sample.

### Effects on Taxa Abundance

**FIGURE 4:**
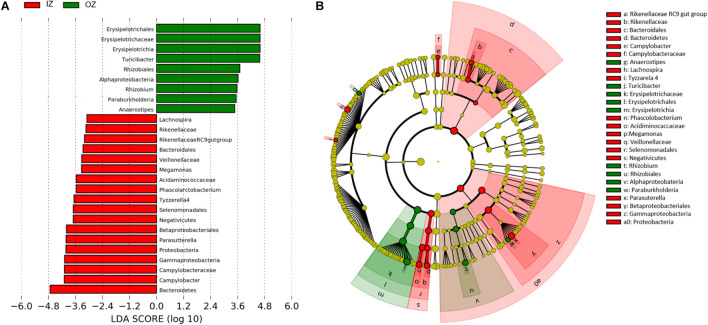
Graphical plot of LEfSe results. **(A)** Histogram of the linear discriminant analysis (LDA) scores computed for features (bacterial groups) differentially abundant in fecal samples of dogs fed diets supplemented with inorganic (IZ and IZ +) or organic (OZ and OZ +) Zn sources without (IZ and OZ) or with (IZ + and OZ +) the addition of a multi-enzymatic complex from the solid-state fermentation of *Aspergillus niger*. **(B)** Taxonomic representation of statistically and biologically differences between fecal samples of dogs fed OZ and OZ + (green) and IZ and IZ + (red) diets; yellow dots are non-significant features (taxa).

The LEfSe was employed to identify the bacterial groups that, independent of their relative abundance, were significantly affected by Zinc or Enzyme, considering the hierarchical relationships inherent in 16S-based taxonomies. Setting an LDA score > 3, 27 taxa were differentially abundant according to zinc source (Figure 4) and none according to enzyme addition (data not shown). At the genus level, Rikenellaceae RC9 gut group, *Parasutterella*, *Lachnospira*, *Megamonas*, *Phascolarctobacterium*, *Tyzzerella* 4, and *Campylobacter* were detected as markers for IZ diets, whereas *Turicibacter*, *Anaerostipes*, *Paraburkholderia*, and *Rhizobium* as markers for OZ diets. At class and phylum levels, Negativicutes, Gammaproteobacteria, Proteobacteria, and Bacteroidetes were marked for IZ diets, and Erysipelotrichia and Alphaproteobacteria for OZ diets.

A further analysis using an NBGLM (Table 3; Supplementary Table 6) enabled the identification of effects of Enzyme and Zinc × Enzyme and confirmed the effect of Zinc obtained with LEfSe with the only exception being the genus *Phascolarctobacterium*, which was similar among zinc sources (*p* = 0.193). In addition to the taxa previously identified by LEfSe, the NBGLM revealed that genera Coriobacteriaceae-002 (*p* = 0.035), *Parabacteroides* (*p* = 0.022), *Peptococcus* (*p* = 0.040), *Erysipelatoclostridium* (*p* = 0.049), and *Butyricicoccus* (*p* = 0.023) were also affected by Zinc, being more abundant in feces of dogs fed inorganic zinc, except for *Butyricicoccus*, which was less abundant (*p* = 0.023).

**TABLE 3:** Most abundant bacterial genera (−log copies) in each phylum extracted from feces of dogs fed diets supplemented with inorganic (IZ and IZ +) and organic (OZ and OZ +) Zn sources without (IZ and OZ) and with (IZ + and OZ +) the addition of a multi-enzymatic complex from the solid-state fermentation of *Aspergillus niger.*

Taxa	Diets^1^				SEM^2^	*p*-Value		
	IZ	IZ +	OZ	OZ +		Zn	Enzyme	Zn × Enzyme
p_Actinobacteria	4.41^ab^	4.28^b^	4.29^b^	4.87^a^	0.156	0.148	0.172	0.029
g_Coriobacteriaceae UCG-002	1.34	1.31	0.28	0.63	0.372	0.035	0.676	0.596
p_Bacteroidetes	8.79	8.84	8.25	8.6	0.177	0.035	0.293	0.392
g_*Alloprevotella*	6.76^a^	6.58^a^	5.84^b^	6.71^a^	0.232	0.108	0.165	0.031
g_*Paraprevotella*	2.07^a^	1.47^a^	0.27^b^	1.66^a^	0.372	0.037	0.324	0.015
g_Rikenellaceae RC9 gut group	2.53	2.31	1.40	1.66	0.372	0.025	0.954	0.538
g_*Parabacteroides*	3.20	3.44	2.57	2.58	0.307	0.022	0.706	0.705
p_Firmicutes	8.08	7.94	8.3	8.35	0.084	0.001	0.609	0.264
g_*Lactobacillus*	4.97	5.34	4.29	5.24	0.313	0.244	0.048	0.373
g_[*Eubacterium*] *brachy* group	3.97^a^	3.41^ab^	2.89^b^	3.59^a^	0.244	0.088	0.789	0.013
g_[*Ruminococcus*] *gnavus* group	2.98^ab^	2.52^b^	2.92^b^	3.52^a^	0.204	0.032	0.729	0.015
g_L*achnoclostridium*	2.66^ab^	2.72^ab^	2.27^b^	3.07^a^	0.163	0.890	0.014	0.032
g_*Lachnospira*	3.96	4.05	1.96	2.46	0.517	0.004	0.577	0.696
g_*Peptococcus*	3.09	2.82	2.10	2.53	0.285	0.040	0.802	0.217
g_*Butyricicoccus*	2.00	2.15	2.58	2.57	0.207	0.023	0.766	0.708
g_Ruminococcaceae UCG-014	3.10^b^	2.33^b^	3.28^b^	4.57^a^	0.360	0.002	0.471	0.016
g_*Erysipelatoclostridium*	3.35	3.47	3.07	2.95	0.191	0.049	0.974	0.541
g_*Holdemanella*	1.66^ab^	1.34^b^	1.19^b^	2.17^a^	0.272	0.530	0.219	0.019
g_*Turicibacter*	5.34	5.03	6.66	6.41	0.307	<0.001	0.398	0.935
p_Proteobacteria	6.60	6.90	6.08	6.40	0.138	<0.001	0.033	0.923
g_*Rhizobium*	1.64	1.33	2.98	2.80	0.510	0.031	0.641	0.894
g_*Parasutterella*	6.34	6.72	5.19	5.77	0.278	0.001	0.099	0.709
p_Tenericutes	0.19^c^	1.55^ab^	1.96^a^	0.34^bc^	0.475	0.560	0.788	0.006
g_*Anaeroplasma*	0.20^b^	1.48^ab^	1.90^a^	0.18^b^	0.492	0.694	0.673	0.006

*Only taxa significantly affected by diets are represented. ^a–c^Values in the same row that share a common superscript are not statistically different (p > 0.05). Letters before bacterial groups designate taxa: p_: phylum, g_: genus.*

*^1^Twelve replicas per treatment.*

*^2^Standard error of the mean.*

Enzyme was associated with higher counts of genus *Lactobacillus* (*p* = 0.048). The phyla Actinobacteria (*p* = 0.029) and Tenericutes (*p* = 0.006), the genera *Alloprevotella* (*p* = 0.031) and *Paraprevotella* (*p* = 0.015), [*Eubacterium*] *brachy* group (*p* = 0.013), [*Ruminococcus*] *gnavus* group (*p* = 0.015), *Lachnoclostridium* (*p* = 0.032), Ruminococcaceae UCG-014 (*p* = 0.016), *Holdemanella* (*p* = 0.019), and *Anaeroplasma* (*p* = 0.006) were significantly affected by Zinc × Enzyme, with similar counts in dogs fed IZ and IZ +, whereas there was a higher number in dogs fed OZ + compared with OZ, except for *Anaeroplasma* for which the contrary was observed.

### Fecal pH, Ammonia-N, Lactate, and Volatile Fatty Acids

Fecal pH, ammonia-N and lactate contents, total VFA production, and individual VFA concentrations are presented in Table 4. None of the fermentation parameters were affected by Zinc, Enzyme, or Zinc × Enzyme (*p* > 0.05).

**TABLE 4:** pH, ammonia-N, lactate, and volatile fatty acids (VFA) content and profile of feces collected from dogs fed diets supplemented with inorganic (IZ and IZ +) and organic (OZ and OZ +) Zn sources without (IZ and OZ) and with (IZ + and OZ +) the addition of a multi-enzymatic complex from the solid-state fermentation of *Aspergillus niger.*

	Diets^1^				SEM^2^	*p*-Value		
	IZ	IZ +	OZ	OZ +		Zn	Enzyme	Zn × Enzyme
pH	6.9	6.7	6.6	6.8	0.11	0.363	0.742	0.174
Ammonia-N, mg/g	48.1	57.5	42.7	51.2	3.52	0.813	0.273	0.214
Lactate, mM	2.67	2.90	2.65	4.05	0.822	0.495	0.333	0.484
Total VFA, μmol/g	159	166	170	170	8.0	0.337	0.681	0.667
Acetate, μmol/g	87.4	92.3	94.9	93.4	4.17	0.305	0.690	0.447
Propionate, μmol/g	40.8	44.2	46.6	47.1	3.29	0.199	0.556	0.676
*Iso*-butyrate, μmol/g	2.08	2.21	2.20	2.53	0.191	0.265	0.245	0.604
Butyrate, μmol/g	22.5	20.7	20.6	20.6	2.39	0.683	0.722	0.716
*Iso*-valerate, μmol/g	2.59	2.84	2.80	3.12	0.157	0.140	0.080	0.817
Valerate, μmol/g	2.24	2.14	1.96	1.86	0.424	0.513	0.827	0.998
*Iso*-caproate, μmol/g	0.99	0.97	0.81	1.05	0.096	0.598	0.258	0.187
Caproate, μmol/g	0.19	0.22	0.21	0.20	0.020	0.807	0.517	0.386
Heptanoate, μmol/g	0.04	0.04	0.04	0.03	0.004	0.772	0.868	0.246

*^1^Twelve replicas per treatment.*

*^2^Standard error of the mean.*

### DNA Abundance of Selected Bacteria

The abundance of selected bacteria (Table 5) was significantly affected by Zinc. *Clostridium* cluster I (*p* = 0.028), *Clostridium* cluster XIV (*p* = 0.006), *Campylobacter* (*p* = 0.013), Ruminococcaceae (*p* = 0.012), *Turicibacter* (*p* = 0.014), and *Blautia* (*p* = 0.024) were higher in feces of dogs fed inorganic zinc. In turn, Enzyme and Zinc × Enzyme did not affect the abundance of selected bacteria (*p* > 0.05).

**TABLE 5:** Log_10_ DNA copies per g of feces collected from dogs fed diets supplemented with inorganic (IZ and IZ +) and organic (OZ and OZ +) Zn sources without (IZ and OZ) and with (IZ + and OZ +) the addition of a multi-enzymatic complex from the solid-state fermentation of *Aspergillus niger*.

	Diets^1^				SEM^2^	*p*-Value		
	IZ	IZ +	OZ	OZ +		Zn	Enzyme	Zn × Enzyme
Clostridium cluster I	9.00	8.84	8.59	8.54	0.152	0.028	0.485	0.726
Clostridium cluster XIV	9.57	9.60	9.13	9.13	0.151	0.006	0.913	0.914
*Campylobacter* spp.	6.96	6.86	5.92	5.46	0.460	0.013	0.549	0.689
*Campylobacter jejuni*	2.92	3.29	3.34	2.31	0.694	0.725	0.655	0.321
*Campylobacter lari*	4.72	4.58	4.33	2.87	0.542	0.062	0.151	0.232
*Escherichia coli*	6.80	6.56	6.47	6.42	0.234	0.333	0.539	0.689
Ruminococcaceae	9.03	9.19	8.66	8.61	0.179	0.012	0.788	0.569
*Turicibacter* spp.	9.76	9.65	9.45	9.54	0.090	0.014	0.890	0.227
*Blautia* spp.	8.60	8.67	8.30	8.22	0.158	0.024	0.962	0.621

*^1^Twelve replicas per treatment.*

*^2^Standard error of the mean.*

## Discussion

The effects of zinc sources on the gut microbiota of dogs remain unclear; and therefore, the present study seeks to provide further insights on the topic. Exogenous enzymes were also tested, as it was surmised that they could improve the bioavailability of zinc, particularly zinc sulfate, by degrading components that may interfere with the bioavailability of minerals (e.g., phytates). Moreover, it was expected that by fermenting undigested nutrients and enhancing the digestibility of diets, enzymes could potentially affect the fecal microbiota of dogs. Indeed, it was observed that bacterial composition was affected differently with either zinc sulfate or zinc proteinate, supplemented at similar levels. However, the enzyme addition and interaction between enzyme addition and zinc source exerted a modest effect on the bacterial composition.

### Effect of Zinc Source

Both Shannon’s diversity index, which summarizes taxonomic richness and evenness, and Faith’s PD, an index of phylogenetic diversity, were not affected by Zinc. PERMANOVA was used to test for differences in beta diversity (Anderson, 2017). As PERMANOVA is known to be sensitive to differences in dispersion among groups, PERMDISP test, a non-parametric multivariate equivalent to a traditional Levene’s test, was conducted to test for homogeneity of dispersions, thus giving insight on within- and between-group dispersion to enable more accurate interpretation of the PERMANOVA (Anderson, 2017). PERMANOVA on unweighted and weighted UniFrac distances indicated a significant effect of Zinc. However, the significant PERMDISP results showed that the multivariate dispersions were not homogenous between zinc sources, with greater dispersion being observed among organic than inorganic zinc supplemented diets. Being both PERMANOVA and PERMDISP significant, a clear dispersion effect exists, not being clear the existence of Zinc effect. The differential impact of zinc sources on the dispersion of samples was, however, not possible to elucidate through the present study.

Compared with the organic source, inorganic zinc was associated with enrichment of Bacteroidetes phylum, including the Rikenellaceae family (genus Rikenellaceae RC9 gut). In dogs, Rikenellaceae was associated with the consumption of high-fat diets (Moinard et al., 2020), which suggests its involvement in lipid metabolism. In mice, the Rikenellaceae RC9 gut group was positively correlated with acetate and butyrate production (Wang et al., 2020). Despite the significantly higher fat intake with inorganic zinc diets (reflecting their slightly higher fat content and dry matter (DM) intake) (complementary data previously published; Pereira et al., 2020), fecal acetate and butyrate concentrations remained unaffected. Within the phylum Actinobacteria, Coriobacteriaceae-002 was more abundant in feces from dogs fed inorganic zinc. Similarly, the involvement of Coriobacteriaceae in lipid metabolism has been suggested (Beloshapka et al., 2013), and the slightly higher fat intake of dogs fed inorganic zinc might contribute to explain these results.

The phylum Proteobacteria and its genus *Parasutterella* (class Gammaproteobacteria) were identified as markers of inorganic zinc supplementation. This agrees with a study in broilers, in which inorganic zinc (zinc oxide) promoted an increase of several genera belonging to the phylum Proteobacteria compared with organic zinc (amino acid chelate; De Grande et al., 2020). Although Proteobacteria are the third or fourth most abundant phylum in the gut of healthy dogs (Suchodolski, 2016; Jha et al., 2020), a significant increase of this phylum was associated with dysbiosis by either infectious (Park et al., 2019) or idiopathic inflammatory diseases (Guard and Suchodolski, 2016). In the present study, there were no gastrointestinal clinical signs that could be related to dysbiosis, and the increase of Proteobacteria was far from the values reported in intestinal diseases. Zinc has been reported effective in controlling enteropathogenic *E. coli* in humans (Crane et al., 2007). Also, a study in mice showed that treatment with zinc attenuated α-hemolysin *E. coli*-induced barrier defects in the colon, decreasing bacterial translocation and antigen stimulation through the compromised epithelial barrier, characteristic of infections and inflammatory bowel diseases (Wiegand et al., 2017). Therefore, the fecal DNA of *E. coli* was quantified by qPCR. Results showed no effect of zinc source on fecal *E. coli* of healthy dogs; however, it does not exclude differential effects of inorganic and organic zinc in case of disease, and therefore, clinical studies are needed.

Another marker of inorganic zinc supplementation was the enrichment in *Campylobacter* (class Epsilonproteobacteria), an important enteropathogen for dogs. However, it was identified in only seven of the 48 fecal samples analyzed, with a relative abundance that did not surpass 0.1%, and was not associated with signs of gastrointestinal disease. Indeed, Acke (2018) detected *Campylobacter* in 56% of healthy dogs’ feces and a greater prevalence (97%) in feces of diarrheic dogs. As zinc controls bacterial gene expression, being responsible for general cellular metabolism and acting as a cofactor of virulence factors (Kehl-Fie and Skaar, 2010), bacteria such as *Campylobacter* spp. need it to grow and infect hosts. In broilers, competition between *C. jejuni* and hosts was observed, even limiting zinc availability for hosts (Gielda and DiRita, 2012). It is not clear whether bacteria can successfully uptake both free and chelated zinc (Hood and Skaar, 2012) nor the amount of chelate that remains bond when it reaches the small and large intestine. To further investigate the enrichment of *Campylobacter* with inorganic zinc, the DNA of *Campylobacter* spp., *C. jejuni*, and *C. lari* was quantified by qPCR. Higher *Campylobacter* spp. and *C. lari* DNA in the feces of dogs fed inorganic zinc suggest that this might be a preferred source for bacteria, which may have an impact on both the host and the microbiota, as both require zinc for physiological functions. This finding is relevant, on the one hand, because microorganisms might be limiting the bioavailability of zinc for the host and, on the other hand, because inorganic zinc supplementation potentially promotes putative pathogenic bacteria such as *Campylobacter*. Also, it is possible that for being more bioavailable, the absorption of zinc proteinate may be higher in the small bowel, hindering it from reaching the colon to be used by bacteria. Indeed, this idea is supported by a study by De Grande et al. (2020), which reported a lower excretion of zinc in broilers fed a diet supplemented with organic zinc (amino acid chelate) in comparison with zinc sulfate.

In contrast, two genera markedly higher in dogs fed organic zinc were *Rhizobium* (class Alphaproteobacteria) and *Paraburkholderia* (class Betaproteobacteria). Rhizobiales are symbiotic in root plants, despite some genera and species being adapted to live in host mammals (Kosoy and Goodrich, 2018). Genus *Rhizobium* includes opportunistic bacteria associated with clinical manifestation in immunocompromised humans. Although already identified in fecal samples of dogs, there is no available information concerning its pathogenicity in dogs (Tabar et al., 2010). Based on pathogenicity, genus *Burkholderia* was divided into two taxa, and clinically relevant and phytopathogenic species were assigned to *Burkholderia*, whereas primary environmental species were transferred to *Paraburkholderia* (Sawana et al., 2014). Therefore, according to this classification, the genus observed in dogs’ feces is considered non-pathogenic. Furthermore, the presence of these microorganisms might be due to undesirable contamination, as dogs have access to grassed or planted areas during daily walks.

Within phylum Firmicutes, most of the microorganisms affected by Zinc are positively related to the production of VFA. For instance, *Lachnospira*, *Tyzzerella* 4 (both increased with inorganic zinc), and *Anaerostipes* (increased with organic zinc) belong to the Lachnospiraceae family, known to ferment complex carbohydrates to VFA (Rodrigues et al., 2016). *Turicibacter*, which was increased with organic zinc, ferments organic matter produced by the Krebs cycle into acetate and succinate (Wu et al., 2017). *Peptococcus*, which was more abundant in feces of dogs fed inorganic zinc, and *Butyricicoccus*, of low abundance in feces of dogs fed inorganic zinc, were both positively correlated with butyrate in dogs’ feces (Sandri et al., 2017; Xu et al., 2019). The VFA is an energy source for enterocytes, can also promote mucosal integrity, and regulate immune responses of the host (Tungland, 2018). Despite the changes in fecal bacterial composition here observed associated with zinc source, those were not reflected in differences on the fecal VFA concentration or other fermentation products (lactate and ammonia), or pH. However, the gut and its communities encompass highly complex interactions between microorganisms; and between these and the host, and the concentration of fecal fermentation products might not reveal the whole picture. A deeper and complementary metagenomic analysis could provide further insights into microbial activity, and thus it is suggested for future work.

The association of the zinc status and dogs’ behavior has been observed (Soltanian et al., 2016). Interestingly, the abundance of *Megamonas* and *Phascolarctobacterium* in dogs’ feces, which responded to Zinc, was previously reported to be, respectively, negatively and positively associated with aggressive and phobic behaviors (Mondo et al., 2020). Despite that in the present study dogs did not exhibit a poor zinc status, nor relevant behavior differences, further studies could elucidate a potential connection of zinc status, microbiota, and inappropriate dog behavior.

Inorganic zinc promoted a higher DNA abundance of *Blautia*, *Turicibacter*, Ruminococcaceae, and *Clostridium* cluster XIV, presumably beneficial due to its higher abundance in the gut of healthy individuals compared with dogs with intestinal diseases (Alessandri et al., 2020), as well as *Clostridium* cluster I that includes the potential pathogen, *C. perfringens*. As inorganic zinc promoted both beneficial and potential pathogenic bacteria and considering that the modulation was not associated with differences in the fecal metabolites herein evaluated nor health status of dogs, the implications for the health of the host remain unclear. However, these results may suggest differences in the bioavailability of zinc sources. According to Suchodolski et al. (2008), *Clostridium* cluster XIV is more abundant in the colon than in the small bowel of dogs. Therefore, one may hypothesize that because it is less bioavailable, inorganic zinc would reach the colon in higher abundance, which is also supported by a study by Starke et al. (2014), who observed that *Clostridium* cluster XIVa was positively correlated with free zinc in the hindgut of weaned piglets. Ruminococcaceae was found to have increased in cecal microbiota of chicks fed zinc-deficient diets (Reed et al., 2015) and in fecal samples with limited zinc supply (Sauer and Grabrucker, 2019). As Ruminococcaceae is also more abundant in the large than small intestine of dogs (Honneffer et al., 2017), this seems to contradict the previous hypothesis, because, in a deficient diet, less zinc would reach the colon, since regulation of intestinal absorption of zinc is an efficient mechanism of homeostasis in healthy individuals. However, the distribution of taxa in the intestinal tract might differ between dogs, mice, and broilers. Also, zinc deficiency has other implications for intestinal microbiota and animal health, which possibly affect Ruminococcaceae, rather than a difference in zinc availability for bacteria. To the best of the authors’ knowledge, the biogeography of *Blautia*, *Turicibacter*, and *Clostridium* cluster I along the intestinal segments of dogs has not been reported, which could have been useful to find a relation between zinc availability and bacterial abundance. Furthermore, the mechanisms of bacterial use of zinc are still developing and will be important to better understand if bioavailability is an important factor for gut bacteria and how it might affect the host.

### Effects of Enzyme Addition

The addition of enzymes did not affect Shannon’s diversity index but significantly affected Faith’s PD index, thus suggesting that species found in the feces of dogs fed diets supplemented with enzyme had more distant phylogenetic relationships. Enzyme addition increased the abundance of genus *Lactobacillus*. A study in broilers showed that adding xylanase to diets increased the abundance of *Lactobacillus* in ileal content (Machado et al., 2020). The addition of carbohydrase can promote degradation of dietary non-starch polysaccharides, with an increase of lactate, organic acids, and VFA and lower ammonia-N in chicks’ excreta (Yadav and Jha, 2019). The fecal concentration of lactate was similar with enzyme addition, despite genus *Lactobacillus* being a major producer of lactate. This is likely explained by the adaption of lactate-consuming species that, facing an increased supply of lactate, can normalize lactate levels, which can happen in 14 days, according to a study by Swanson et al. (2002). Even though the interaction of microorganisms is complex, it was observed that *Lachnoclostridium*, a lactate consumer that is stimulated by the presence of lactate producers such as *Lactobacillus* (Gutiérrez and Garrido, 2019), was increased with the addition of enzyme, which supports the idea that lactate results might be related to cross-feeding.

Phylum Proteobacteria was higher with enzyme addition; however, an effect on specific members of this phylum was not observed. Thus, the result is not explained by the response of a core family or genus of this phylum but likely by underrepresented species.

Tortola et al. (2013) evaluated high and low levels of enzyme addition (containing protease, cellulase, pectinase, phytase, β-glucanase, and xylanase), reporting effects on total VFA production, acetate, and butyrate contents, using the higher level. Similarly, De Smet et al. (1999) reported an increase in diet digestibility and fecal VFA and lactate contents associated with the addition of a high level of phytase and α-galactosidase, as well as different outcomes when enzymes were added separately. In turn, Sá et al. (2013) reported absence of effects on the proportion of fecal VFA of dogs with the addition of an enzymatic complex containing a level of enzymes (β-glucanase, xylanase, cellulase, glucoamylase, phytase, and α-amylase) below the ones used by Tortola et al. (2013). Similarly, in the present study, the enzymes showed no effects on the end-products of fecal fermentation, agreeing with the lack of effects on the digestibility of diets reported by Pereira et al. (2020). Contrasting reports may be explained by differences in the amount and/or activity of enzymes added, which are not always made available, precluding a direct comparison between studies.

### Effect of Interaction Between Zinc Source and Enzyme Addition

The addition of enzymes was associated with an increase of some taxa of the phyla Actinobacteria and Firmicutes in feces of dogs fed organic zinc but not with the inorganic source. The abundance of Actinobacteria was found to be higher in obese dogs compared with lean dogs (Handl et al., 2013). However, in the present study, the body condition score averaged 4.5 (on a 9-point scale), with no differences being observed between consecutive periods of 35 days each (Pereira et al., 2020). Higher dietary fiber levels have been also shown to increase the abundance of species belonging to phyla Actinobacteria and Firmicutes (Deehan et al., 2017; Li et al., 2017). Even though these species contain few fibrolytic enzymes, they are considered to play an important role in the initiation of substrate degradation (Makki et al., 2018). In the present study, the ingredient composition of diets only differed on the zinc source and the addition of a multi-enzymatic complex. However, the digestibility of neutral detergent fiber (composed of cell wall constituents) was affected by the interaction between zinc source and enzyme addition, the lowest and highest values being observed for IZ and IZ + diets, respectively, with OZ and OZ + not differing from the other treatments (Pereira et al., 2020).

The abundance of genera *Alloprevotella* and *Paraprevotella* was significantly lower in feces from dogs fed the OZ comparing to IZ, IZ +, and OZ +. *Alloprevotella* is one of the main genera identified in the dog microbiome (Kubinyi et al., 2020), and studies, in dogs and other animal species, have shown that the dietary ratio of soluble and insoluble fiber can selectively regulate its intestinal abundance (Chen et al., 2019; Sandri et al., 2019). However, as already stated, fiber content and type used in the present study did not differ between dietary treatments. Moreover, despite *Paraprevotella* having been shown to positively correlate with propionate concentration (Mayengbam et al., 2019), no differences were observed in VFA production or profile.

## Conclusion

Zinc sources affected the fecal bacterial composition of dogs, which was reflected in the abundance of bacterial groups at several taxonomic levels. It was observed that the DNA abundance of the selected bacterial groups was higher in the feces of dogs that received diets supplemented with inorganic zinc, compared with organic zinc. This suggests differences in the bioavailability of zinc, although knowledge on requirements and differential bacterial use of zinc sources is still limited. A higher abundance of genus *Lactobacillus* was observed in dogs fed enzyme-supplemented diets. The abundance of some taxa of the phyla Actinobacteria and Firmicutes was affected by the addition of enzymes to organic zinc. Further studies are required to unveil the effects of the interaction between zinc sources and enzyme addition on the fecal microbial community, as well as the implications for the host (dog).

## Data Availability Statement

Raw sequences of 16S rRNA gene are available at NCBI, accession number PRJEB44253.

## Ethics Statement

The animal study was reviewed and approved by the Local Animal Ethics Committee of Abel Salazar Biomedical Sciences Institute, University of Porto, and licensed by the Portuguese Directorate-General of Food and Veterinary Medicine (permit N.° 206/2017). All the procedures were performed by trained personnel (FELASA category C).

## Author Contributions

AF and AC designed the study. EM supplied the experimental diets. AP, AC, and MM conducted the trial and collected samples. AP and MM run end-fermentation product analysis. AP and CP performed the DNA extraction and the qPCR essays. AP, MM, CP, GB, AF, and AC analyzed and interpreted data. AP, MM, and AC wrote the first draft of the manuscript. MS revised the manuscript. All authors read and approved the submitted version.

## Conflict of Interest

AP’s Ph.D. scholarship was co-funded by Alltech Portugal. Alltech Portugal approved the publication of the manuscript without interfering in any stage of the study, including experimental design, analysis, and report of the results. EM was employed by the company Sorgal, which produced the diets according to the design of the study. The remaining authors declare that the research was conducted in the absence of any commercial or financial relationships that could be construed as a potential conflict of interest.

## Publisher’s Note

All claims expressed in this article are solely those of the authors and do not necessarily represent those of their affiliated organizations, or those of the publisher, the editors and the reviewers. Any product that may be evaluated in this article, or claim that may be made by its manufacturer, is not guaranteed or endorsed by the publisher.
