# Supplementary material for: Effects of Zinc Source and Enzyme Addition on the Fecal Microbiota of Dogs
Source: Front Microbiol. 2021 Oct 13;12:688392. doi: 10.3389/fmicb.2021.688392 (PMC8549731; doi:10.3389/fmicb.2021.688392)
Supplement: Supplementary file 1 [file Data_Sheet_1.docx]

Supplementary Material


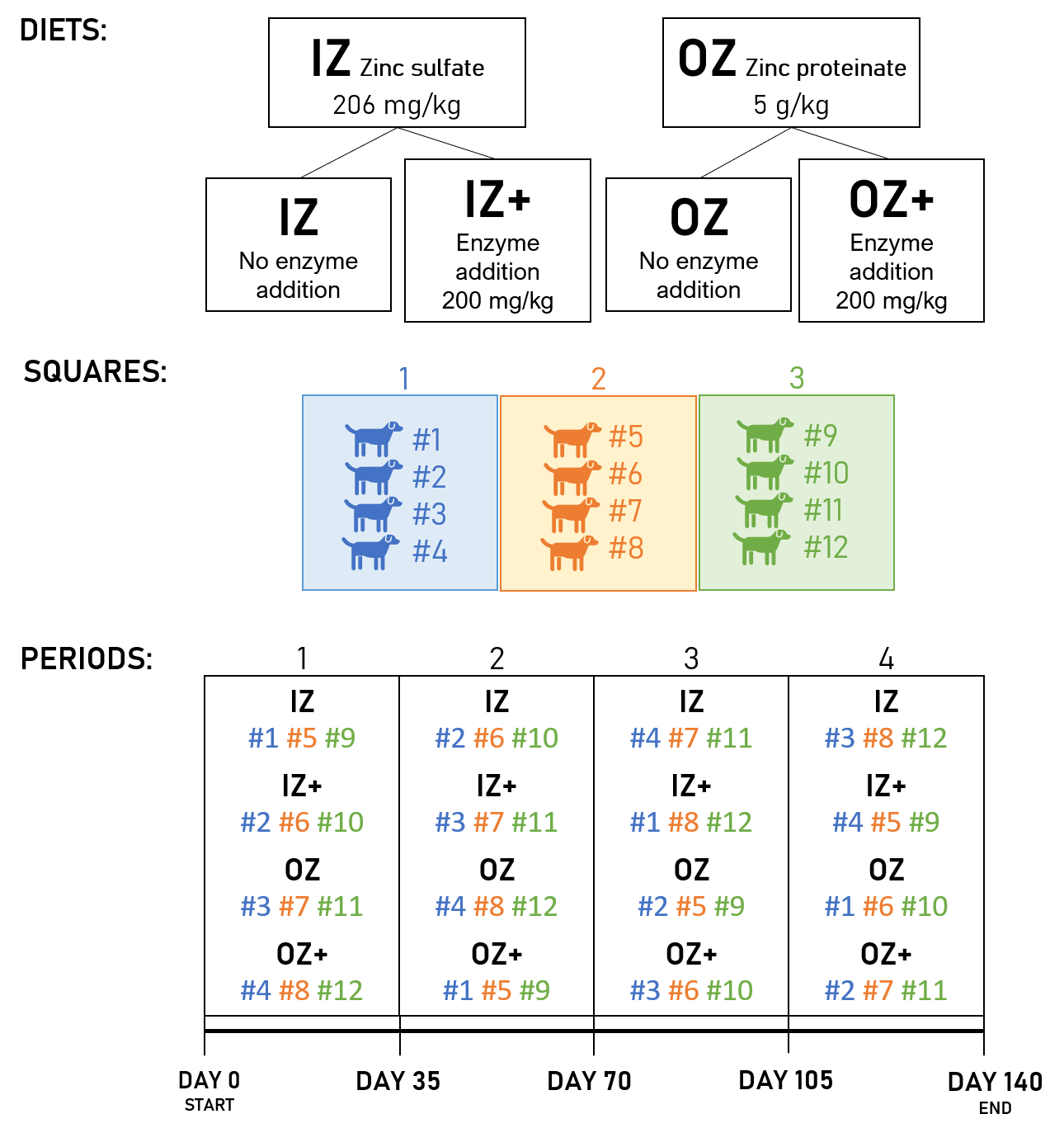


Supplementary Figure 1. Illustration of the experimental design, composition of diets, and squares (n=12 dogs). Assignment of diets to dogs in the 4 periods. The days indicated correspond to the last of each period, in which fresh feces (within 1 h of defecation) were collected for microbiota and end-fermentation product analysis. Enzyme refers to a commercial multi-enzymatic complex from the solid-state fermentation product of *Aspergillus niger* (Synergen^®^, Alltech, KY, USA).

| **A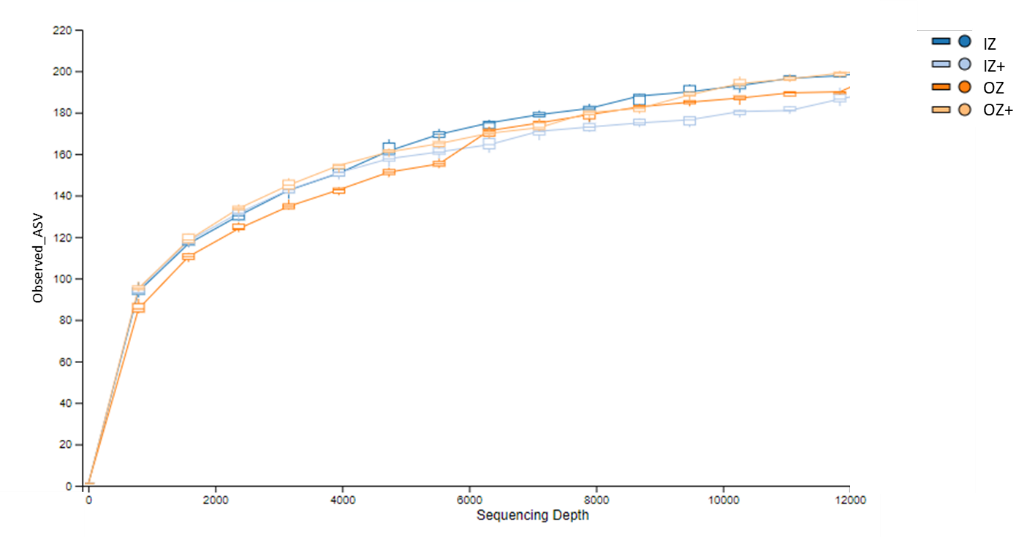** |
| --- |
| **B**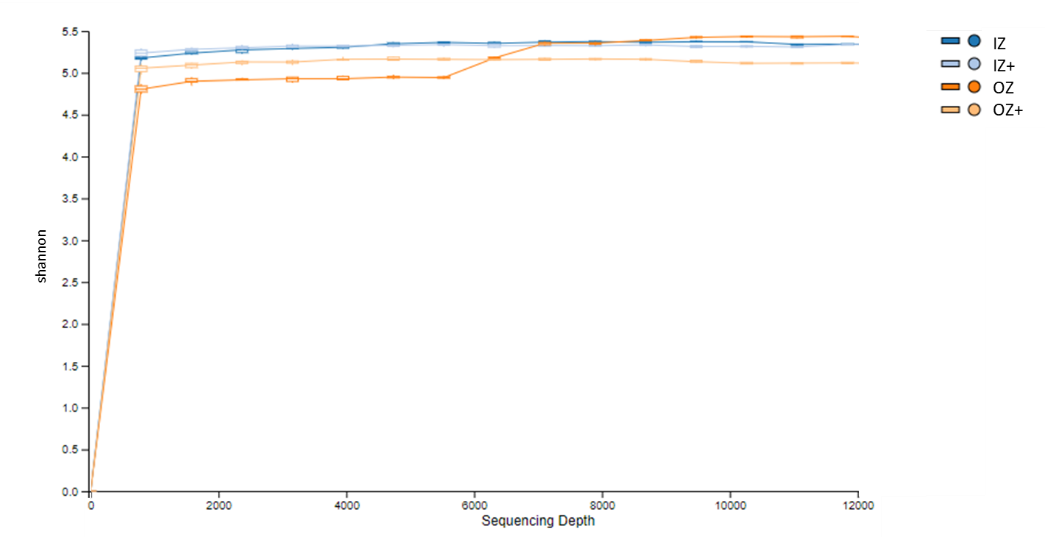 |
| **C**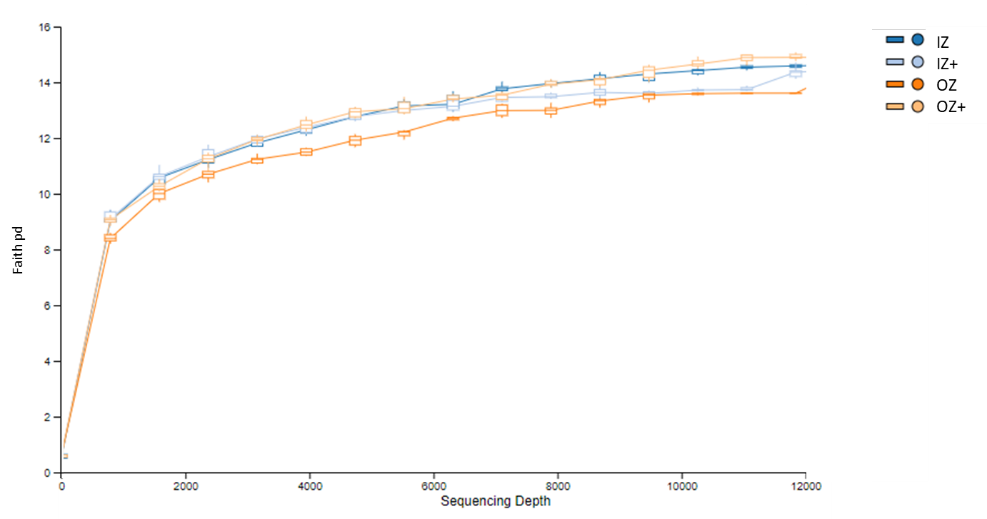 |

**Supplementary Figure 2.** Rarefaction curves of communities in feces of dogs fed the experimental diets. A – Observed ASV’s, B – Shannon’s diversity index, and C – Faith’s phylogenetic diversity. IZ - inorganic Zn in the form of Zn sulfate monohydrate, no enzyme addition; IZ+ - inorganic Zn in the form of Zn sulfate monohydrate with enzyme addition; OZ - organic Zn in the form of a chelate Zn proteinate, no enzyme addition; OZ+ - organic Zn in the form of a chelate Zn proteinate with enzyme addition.


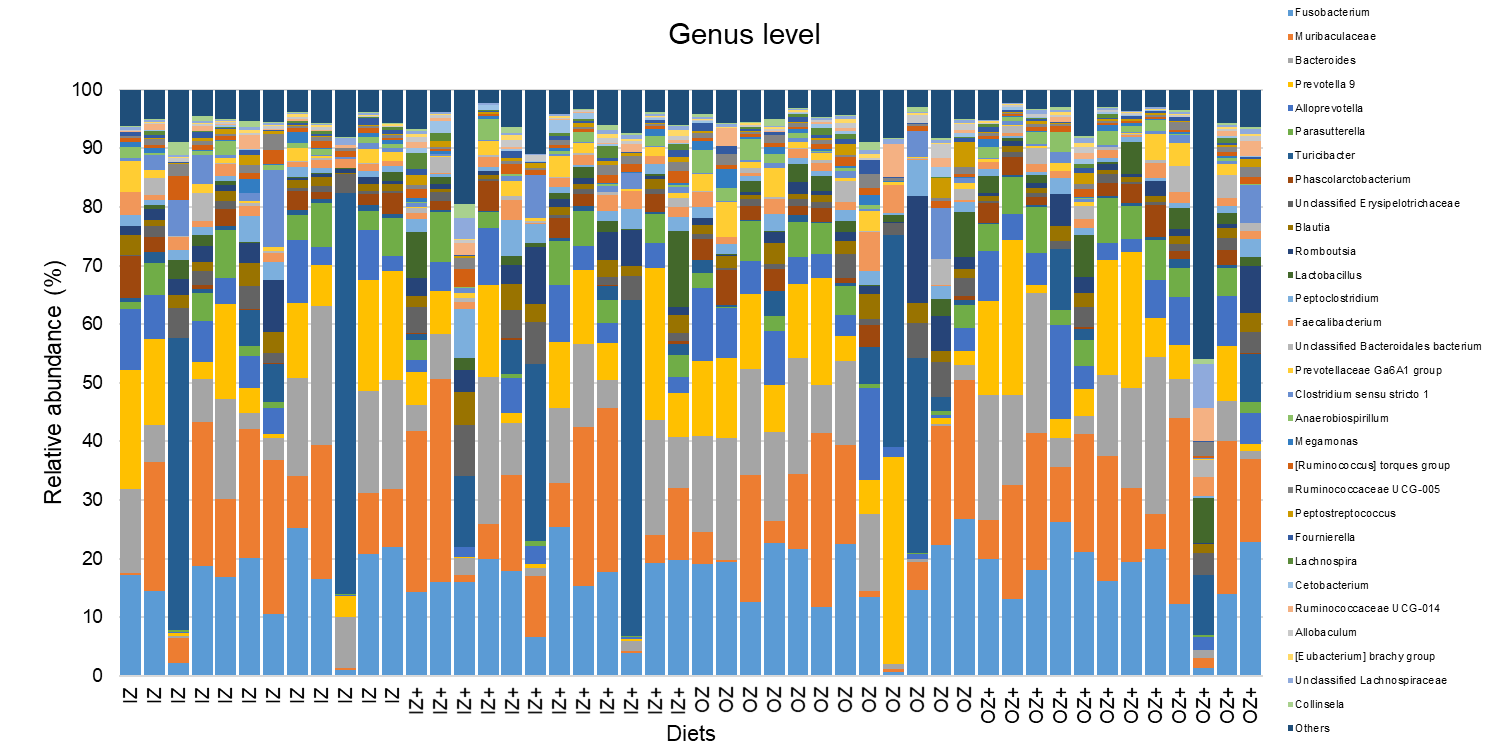


Supplementary Figure 3. Relative abundance (%) of bacterial genera in individual fecal samples of dogs. Genera with relative abundance < 0.5% were pooled and named “Others”. IZ - inorganic Zn in the form of Zn sulfate monohydrate, no enzyme addition; IZ+ - inorganic Zn in the form of Zn sulfate monohydrate with enzyme addition; OZ - organic Zn in the form of a chelate Zn proteinate, no enzyme addition; OZ+ - organic Zn in the form of a chelate Zn proteinate with enzyme addition.

**Supplementary Table 1.** Kruskal-Wallis pairwise comparison of alpha diversity metrics of the fecal microbiome of dogs fed the experimental diets.

| Metrics and diets | | H | *p*-value | *q-*value |
| --- | --- | --- | --- | --- |
| Shannon’s diversity index | | | | |
| IZ (n=12) | IZ+ (n=12) | 0.27 | 0.603 | 0.644 |
| IZ (n=12) | OZ (n=12) | 1.20 | 0.273 | 0.533 |
| IZ (n=12) | OZ+ (n=12) | 0.21 | 0.644 | 0.644 |
| IZ+ (n=12) | OZ (n=12) | 2.61 | 0.106 | 0.533 |
| IZ+ (n=12) | OZ+ (n=12) | 0.96 | 0.326 | 0.533 |
| OZ (n=12) | OZ+ (n=12) | 0.85 | 0.356 | 0.533 |
| Faith’s phylogenetic diversity | | | | |
| IZ (n=12) | IZ+ (n=12) | 0.33 | 0.564 | 0.676 |
| IZ (n=12) | OZ (n=12) | 2.80 | 0.094 | 0.188 |
| IZ (n=12) | OZ+ (n=12) | 1.33 | 0.248 | 0.372 |
| IZ+ (n=12) | OZ (n=12) | 3.41 | 0.065 | 0.188 |
| IZ+ (n=12) | OZ+ (n=12) | 0.00 | 1.000 | 1.000 |
| OZ (n=12) | OZ+ (n=12) | 4.08 | 0.043 | 0.188 |
| Pielou’s Evenness | | | | |
| IZ (n=12) | IZ+ (n=12) | 0.16 | 0.686 | 0.686 |
| IZ (n=12) | OZ (n=12) | 1.76 | 0.184 | 0.553 |
| IZ (n=12) | OZ+ (n=12) | 0.48 | 0.488 | 0.586 |
| IZ+ (n=12) | OZ (n=12) | 2.08 | 0.149 | 0.553 |
| IZ+ (n=12) | OZ+ (n=12) | 0.65 | 0.419 | 0.586 |
| OZ (n=12) | OZ+ (n=12) | 0.65 | 0.419 | 0.586 |

IZ - inorganic Zn in the form of Zn sulfate monohydrate, no enzyme addition; IZ+ - inorganic Zn in the form of Zn sulfate monohydrate with enzyme addition; OZ - organic Zn in the form of a chelate Zn proteinate, no enzyme addition; OZ+ - organic Zn in the form of a chelate Zn proteinate with enzyme addition.

**Supplementary Table 2.** Permutation multivariate analysis PERMANOVA pairwise on Unweighted UniFrac distances in fecal microbiome of dogs fed the experimental diets (Zn × Enzyme interaction).

| Diets | | Sample size | pseudo-F | *p*-value | *q-*value |
| --- | --- | --- | --- | --- | --- |
| IZ | IZ+ | 24 | 0.82 | 0.696 | 0.695 |
| IZ | OZ | 24 | 1.37 | 0.134 | 0.354 |
| IZ | OZ+ | 24 | 1.01 | 0.417 | 0.626 |
| IZ+ | OZ | 24 | 1.60 | 0.061 | 0.354 |
| IZ+ | OZ+ | 24 | 1.26 | 0.177 | 0.354 |
| OZ | OZ+ | 24 | 0.92 | 0.525 | 0.630 |

IZ - inorganic Zn in the form of Zn sulfate monohydrate, no enzyme addition; IZ+ - inorganic Zn in the form of Zn sulfate monohydrate with enzyme addition; OZ - organic Zn in the form of a chelate Zn proteinate, no enzyme addition; OZ+ - organic Zn in the form of a chelate Zn proteinate with enzyme addition.

**Supplementary Table 3.** Permutation multivariate analysis PERMDISP pairwise on Unweighted UniFrac distances in fecal microbiome of dogs fed the experimental diets (Zn × Enzyme interaction).

| Diets | | Sample size | pseudo-F | *p*-value | *q-*value |
| --- | --- | --- | --- | --- | --- |
| IZ | IZ+ | 24 | 0.07 | 0.777 | 0.777 |
| IZ | OZ | 24 | 3.42 | 0.072 | 0.491 |
| IZ | OZ+ | 24 | 0.98 | 0.327 | 0.345 |
| IZ+ | OZ | 24 | 3.18 | 0.115 | 0.345 |
| IZ+ | OZ+ | 24 | 1.18 | 0.304 | 0.491 |
| OZ | OZ+ | 24 | 0.13 | 0.714 | 0.777 |

IZ - inorganic Zn in the form of Zn sulfate monohydrate, no enzyme addition; IZ+ - inorganic Zn in the form of Zn sulfate monohydrate with enzyme addition; OZ - organic Zn in the form of a chelate Zn proteinate, no enzyme addition; OZ+ - organic Zn in the form of a chelate Zn proteinate with enzyme addition.

**Supplementary Table 4.** Permutation multivariate analysis PERMANOVA pairwise on Weighted UniFrac distances in fecal microbiome of dogs fed the experimental diets (Zn × Enzyme interaction).

| Diets | | Sample size | pseudo-F | *p*-value | *q-*value |
| --- | --- | --- | --- | --- | --- |
| IZ | IZ+ | 24 | 0.38 | 0.768 | 0.768 |
| IZ | OZ | 24 | 2.24 | 0.112 | 0.224 |
| IZ | OZ+ | 24 | 1.69 | 0.168 | 0.252 |
| IZ+ | OZ | 24 | 3.25 | 0.061 | 0.192 |
| IZ+ | OZ+ | 24 | 2.69 | 0.064 | 0.192 |
| OZ | OZ+ | 24 | 0.61 | 0.545 | 0.654 |

IZ - inorganic Zn in the form of Zn sulfate monohydrate, no enzyme addition; IZ+ - inorganic Zn in the form of Zn sulfate monohydrate with enzyme addition; OZ - organic Zn in the form of a chelate Zn proteinate, no enzyme addition; OZ+ - organic Zn in the form of a chelate Zn proteinate with enzyme addition.

**Supplementary Table 5.** Permutation multivariate analysis PERMDISP pairwise on Weighted UniFrac distances in fecal microbiome of dogs fed the experimental diets (Zn × Enzyme interaction).

| Diets | | Sample size | pseudo-F | *p*-value | *q-*value |
| --- | --- | --- | --- | --- | --- |
| IZ | IZ+ | 24 | 0.60 | 0.480 | 0.578 |
| IZ | OZ | 24 | 5.69 | 0.059 | 0.177 |
| IZ | OZ+ | 24 | 0.28 | 0.620 | 0.620 |
| IZ+ | OZ | 24 | 9.17 | 0.027 | 0.162 |
| IZ+ | OZ+ | 24 | 1.49 | 0.252 | 0.378 |
| OZ | OZ+ | 24 | 2.85 | 0.117 | 0.234 |

IZ - inorganic Zn in the form of Zn sulfate monohydrate, no enzyme addition; IZ+ - inorganic Zn in the form of Zn sulfate monohydrate with enzyme addition; OZ - organic Zn in the form of a chelate Zn proteinate, no enzyme addition; OZ+ - organic Zn in the form of a chelate Zn proteinate with enzyme addition.

**Supplementary Table 6.** Most abundant bacterial genera (-log copies) in each phylum in feces of dogs fed diets supplemented with inorganic (IZ and IZ+) and organic (OZ and OZ+) Zn sources without (IZ and OZ) and with (IZ+ and OZ+) the addition of a multi-enzymatic complex from the solid-state fermentation of *Aspergillus niger*.

| Taxa | Diets^1^ | | | | SEM^2^ | *p*-value | | |
| --- | --- | --- | --- | --- | --- | --- | --- | --- |
|  | IZ | IZ+ | OZ | OZ+ |  | Zn | Enzyme | Zn×Enzyme |
| p_Actinobacteria | 4.41^ab^ | 4.28^b^ | 4.29^b^ | 4.87^a^ | 0.156 | 0.148 | 0.172 | 0.029 |
| g_*Bifidobacterium* | 3.35 | 3.27 | 2.72 | 3.54 | 0.257 | 0.510 | 0.161 | 0.093 |
| g_*Coriobacteriaceae* UCG-002 | 1.34 | 1.31 | 0.28 | 0.63 | 0.372 | 0.035 | 0.676 | 0.596 |
| g_*Collinsella* | 3.37 | 3.22 | 3.41 | 4.03 | 0.204 | 0.053 | 0.265 | 0.070 |
| g_*Parvibacter* | 1.31 | 1.21 | 1.12 | 1.96 | 0.250 | 0.254 | 0.152 | 0.062 |
| p_Bacteroidetes | 8.79 | 8.84 | 8.25 | 8.6 | 0.177 | 0.035 | 0.293 | 0.392 |
| g_*Bacteroides* | 7.30 | 7.46 | 6.87 | 6.98 | 0.270 | 0.104 | 0.645 | 0.935 |
| g_*Alloprevotella* | 6.76^a^ | 6.58^a^ | 5.84^b^ | 6.71^a^ | 0.232 | 0.108 | 0.165 | 0.031 |
| g_*Paraprevotella* | 2.07^a^ | 1.47^a^ | 0.27^b^ | 1.66^a^ | 0.372 | 0.037 | 0.324 | 0.015 |
| g_*Prevotella* 9 | 7.14 | 7.32 | 6.78 | 6.58 | 0.311 | 0.087 | 0.977 | 0.542 |
| g_*Prevotellaceae* Ga6A1 group | 4.87 | 5.03 | 4.95 | 5.23 | 0.386 | 0.722 | 0.614 | 0.889 |
| g_*Rikenellaceae* RC9 gut group | 2.53 | 2.31 | 1.40 | 1.66 | 0.372 | 0.025 | 0.954 | 0.538 |
| g_*Parabacteroides* | 3.20 | 3.44 | 2.57 | 2.58 | 0.307 | 0.022 | 0.706 | 0.705 |
| p_Deferribacteres | 1.65 | 2.47 | 1.94 | 1.87 | 0.310 | 0.627 | 0.236 | 0.178 |
| g_*Mucispirillum* |  |  |  |  |  |  |  |  |
| p_Epsilonbacteraeota | 2.07 | 2.01 | 3.14 | 1.89 | 0.523 | 0.214 | 0.433 | 0.284 |
| g_*Helicobacter* | 1.72 | 2.97 | 2.08 | 1.83 | 0.576 | 0.510 | 0.455 | 0.209 |
| p_Firmicutes | 8.08 | 7.94 | 8.3 | 8.35 | 0.084 | 0.001 | 0.609 | 0.264 |
| g_*Lactobacillus* | 4.97 | 5.34 | 4.29 | 5.24 | 0.313 | 0.244 | 0.048 | 0.373 |
| g_*CandidatusArthromitus* | 2.73 | 2.47 | 2.37 | 2.61 | 0.453 | 0.800 | 0.983 | 0.574 |
| g_*Clostridiumsensustricto* 1 | 4.84 | 4.28 | 5.17 | 4.9 | 0.278 | 0.105 | 0.150 | 0.623 |
| g_[*Eubacterium*] *brachy* group | 3.97^a^ | 3.41^ab^ | 2.89^b^ | 3.59^a^ | 0.244 | 0.088 | 0.789 | 0.013 |
| g_[*Ruminococcus*] *gauvreauii* group | 2.21 | 2.49 | 2.42 | 2.23 | 0.218 | 0.916 | 0.812 | 0.284 |
| g_[*Ruminococcus*] *gnavus* group | 2.98^ab^ | 2.52^b^ | 2.92^b^ | 3.52^a^ | 0.204 | 0.032 | 0.729 | 0.015 |
| g_[*Ruminococcus*] *torques* group | 4.55 | 4.54 | 4.70 | 4.63 | 0.138 | 0.426 | 0.763 | 0.832 |
| g_*Blautia* | 5.36 | 5.38 | 5.25 | 5.55 | 0.169 | 0.864 | 0.347 | 0.413 |
| g_L*achnoclostridium* | 2.66^ab^ | 2.72^ab^ | 2.27^b^ | 3.07^a^ | 0.163 | 0.890 | 0.014 | 0.032 |
| g_*Lachnospira* | 3.96 | 4.05 | 1.96 | 2.46 | 0.517 | 0.004 | 0.577 | 0.696 |
| g_*Lachnospiraceae* NK4A136 group | 3.52 | 3.79 | 3.18 | 3.62 | 0.205 | 0.232 | 0.096 | 0.699 |
| g_*Marvinbryantia* | 0.80 | 0.92 | 0.62 | 1.42 | 0.281 | 0.569 | 0.101 | 0.239 |
| g_*Oribacterium* | 2.07 | 2.12 | 1.47 | 2.29 | 0.251 | 0.394 | 0.102 | 0.145 |
| g_*Sellimonas* | 2.10 | 1.99 | 2.13 | 2.25 | 0.196 | 0.465 | 0.981 | 0.570 |
| g_*Tyzzerella* | 1.23 | 1.44 | 0.69 | 1.68 | 0.298 | 0.635 | 0.061 | 0.189 |
| g_*Peptococcus* | 3.09 | 2.82 | 2.10 | 2.53 | 0.285 | 0.04 | 0.802 | 0.217 |
| g_*Peptoclostridium* | 5.31 | 5.14 | 5.25 | 5.50 | 0.212 | 0.512 | 0.845 | 0.336 |
| g_*Romboutsia* | 5.42 | 5.18 | 5.62 | 5.73 | 0.242 | 0.139 | 0.789 | 0.483 |
| g_*Terrisporobacter* | 0.75 | 1.26 | 1.58 | 0.55 | 0.503 | 0.908 | 0.622 | 0.156 |
| g_*Anaerofilum* | 3.12 | 3.15 | 2.65 | 2.78 | 0.216 | 0.064 | 0.709 | 0.812 |
| g_*Butyricicoccus* | 2.00 | 2.15 | 2.58 | 2.57 | 0.207 | 0.023 | 0.766 | 0.708 |
| g_*Faecalibacterium* | 5.13 | 5.35 | 5.10 | 5.46 | 0.189 | 0.825 | 0.148 | 0.719 |
| g_*Fournierella* | 4.26 | 4.18 | 3.71 | 4.25 | 0.233 | 0.323 | 0.369 | 0.191 |
| g_*Negativibacillus* | 5.58 | 5.71 | 5.48 | 5.85 | 0.160 | 0.886 | 0.136 | 0.470 |
| g_*Ruminiclostridium* 9 | 0.85 | 1.59 | 0.65 | 0.54 | 0.351 | 0.089 | 0.378 | 0.253 |
| g_*Ruminococcaceae* UCG-005 | 4.27 | 4.42 | 4.47 | 4.72 | 0.137 | 0.076 | 0.167 | 0.718 |
| g_*Ruminococcaceae* UCG-014 | 3.10^b^ | 2.33^b^ | 3.28^b^ | 4.57^a^ | 0.360 | 0.002 | 0.471 | 0.016 |
| g_*Allobaculum* | 3.99 | 3.78 | 3.76 | 3.84 | 0.189 | 0.665 | 0.732 | 0.466 |
| g_*Dubosiella* | 3.21 | 3.20 | 3.18 | 3.25 | 0.218 | 0.972 | 0.902 | 0.873 |
| g_*Erysipelatoclostridium* | 3.35 | 3.47 | 3.07 | 2.95 | 0.191 | 0.049 | 0.974 | 0.541 |
| g_*Holdemanella* | 1.66^ab^ | 1.34^b^ | 1.19^b^ | 2.17^a^ | 0.272 | 0.530 | 0.219 | 0.019 |
| g_*Turicibacter* | 5.34 | 5.03 | 6.66 | 6.41 | 0.307 | <0.001 | 0.398 | 0.935 |
| g_*Phascolarctobacterium* | 5.30 | 5.82 | 4.90 | 4.88 | 0.494 | 0.193 | 0.659 | 0.592 |
| g_*Megamonas* | 4.69 | 4.21 | 3.8 | 3.54 | 0.392 | 0.068 | 0.39 | 0.782 |
| p_Fusobacteria | 7.82 | 7.85 | 7.28 | 7.83 | 0.210 | 0.207 | 0.181 | 0.216 |
| g_*Cetobacterium* | 3.93 | 3.18 | 2.96 | 3.06 | 0.410 | 0.246 | 0.442 | 0.293 |
| g_*Fusobacterium* | 7.79 | 7.83 | 7.26 | 7.82 | 0.209 | 0.222 | 0.165 | 0.215 |
| p_Proteobacteria | 6.60 | 6.90 | 6.08 | 6.40 | 0.138 | <0.001 | 0.033 | 0.923 |
| g_*Rhizobium* | 1.64 | 1.33 | 2.98 | 2.80 | 0.510 | 0.031 | 0.641 | 0.894 |
| g_*Anaerobiospirillum* | 4.40 | 4.97 | 4.27 | 4.29 | 0.400 | 0.338 | 0.475 | 0.508 |
| g_*Parasutterella* | 6.34 | 6.72 | 5.19 | 5.77 | 0.278 | 0.001 | 0.099 | 0.709 |
| g_*Sutterella* | 2.01 | 3.32 | 3.12 | 2.57 | 0.496 | 0.741 | 0.471 | 0.071 |
| g_*Escherichia-Shigella* | 1.38 | 1.68 | 1.44 | 2.50 | 0.464 | 0.362 | 0.157 | 0.450 |
| p_Tenericutes | 0.19^c^ | 1.55^ab^ | 1.96^a^ | 0.34^bc^ | 0.475 | 0.560 | 0.788 | 0.006 |
| g_*Anaeroplasma* | 0.20^b^ | 1.48^ab^ | 1.90^a^ | 0.18^b^ | 0.492 | 0.694 | 0.673 | 0.006 |

a–c Values in the same row that share a common superscript are not statistically different (*p* > 0.05).

Letters before bacterial groups designate taxa: p_: phylum, g_: genus.

^1^Twelve replicas per treatment.

^2^Standard error of the mean.
